# Acupuncture for comorbid depression and insomnia in perimenopause: A feasibility patient-assessor-blinded, randomized, and sham-controlled clinical trial

**DOI:** 10.3389/fpubh.2023.1120567

**Published:** 2023-02-06

**Authors:** Fei-Yi Zhao, Zhen Zheng, Qiang-Qiang Fu, Russell Conduit, Hong Xu, Hui-Ru Wang, Yu-Ling Huang, Ting Jiang, Wen-Jing Zhang, Gerard A. Kennedy

**Affiliations:** ^1^School of Health and Biomedical Sciences, RMIT University, Bundoora, VIC, Australia; ^2^Shanghai Municipal Hospital of Traditional Chinese Medicine, Shanghai University of Traditional Chinese Medicine, Shanghai, China; ^3^Department of Nursing, School of International Medical Technology, Shanghai Sanda University, Shanghai, China; ^4^Yangpu Hospital, School of Medicine, Tongji University, Shanghai, China; ^5^Institute for Breathing and Sleep, Austin Health, Heidelberg, VIC, Australia; ^6^Institute of Health and Wellbeing, Federation University, Mount Helen, VIC, Australia

**Keywords:** perimenopause, depression, insomnia, comorbid depression and insomnia, acupuncture, non-pharmacologic intervention, hormone levels, clinical trial

## Abstract

**Background and objective:**

Whilst acupuncture is widely used for treating psychosomatic diseases, there is little high-quality evidence supporting its application in comorbid perimenopausal depression (PMD) and insomnia (PMI) which are common complaints during climacteric. This feasibility, patient-assessor-blinded, randomized, sham-controlled clinical trial addresses this gap by investigating the efficacy and safety of acupuncture on depressed mood and poor sleep in women with comorbid PMD and PMI.

**Methods:**

Seventy eligible participants were randomly assigned to either real-acupuncture (RA) or sham-acupuncture (SA) groups. Either RA or SA treatment were delivered in 17 sessions over 8 weeks. The primary outcomes for mood and sleep were changes on 17-items Hamilton Depression Rating Scale (HAM-D_17_) and Pittsburgh Sleep Quality Index (PSQI) scores, from baseline to 16-week follow-up. Secondary outcome measures involved anxiety symptoms, perimenopausal symptoms, quality of life, participants' experience of and satisfaction with the acupuncture treatment. Blood samples were taken to measure reproductive hormone levels. Intention-To-Treat and Per-Protocol analyses were conducted with linear mixed-effects models. The James' and Bang's blinding indices were used to assess the adequacy of blinding.

**Results:**

Sixty-five participants completed all treatment sessions, and 54 and 41 participants completed the eight- and 16-week follow-ups, respectively. At post-treatment and 8-week follow-up, the RA group showed a significantly greater reduction in PSQI scores than the SA group did; although the reduction of HAM-D_17_ scores in RA group was significant, the change was not statistically different from that of SA. There were no significant mean differences between baseline and 16-week follow-up in either HAM-D_17_ or PSQI in either group. There were no significant between-group differences in serum reproductive hormone levels. All treatments were tolerable and no serious adverse events were reported, and the blinding was successful.

**Conclusion:**

Acupuncture is safe and can contribute to clinically relevant improvements in comorbid PMD and PMI, with satisfactory short-and medium-term effects. Whether the anti-depressive benefit of acupuncture is specific or non-specific remains to be determined. No evidence was found for any longer-term benefit of acupuncture compared to sham at 16 weeks. Further research is required to elucidate mechanisms underlying the short to medium term effects of acupuncture.

## 1. Introduction

Perimenopausal depression (PMD) manifests as depressed mood, reduced happiness, despair, and even suicidal ideation (1). PMD is also the leading cause of disease-related disability in perimenopausal women (2). Depression and insomnia usually co-occur, and comorbidity is associated with poorer outcomes for both conditions (3) and can be more difficult to treat (4). Beyond adverse effects on life quality and occupational performance, PMD and PMI are associated with increased healthcare costs (5, 6). The prevalence of comorbid PMD and PMI are around 31.5% (7). In addition, PMD and PMI have a strong bidirectional relationship (8). Comorbidity also confers a greater risk of relapse of depression (4).

Cognitive behavioral therapy (CBT) is an effective front-line treatment for PMD comorbid with/without PMI (9). The primary problem with CBT is access to treatment due to a lack of qualified practitioners (10) and long waiting times (11). Sedating-antidepressant agent (e.g., Mirtazapine, Trazodone, Agomelatine) are a reasonable option for treating comorbid depression and insomnia (12). However, these psychotropic agents can create dependence and also have adverse effects (AEs) causing dry mouth, nausea, dizziness, increased appetite and rapid weight gain, constipation, and sexual dysfunction (13). Hormonal replacement therapy (HRT) has been a routine and effective strategy for the management of perimenopausal symptoms including PMD and PMI (14, 15). However, many women may reject HRT due to concerns about potential risks associated with cancer of breasts, uterus and ovaries.

Complementary and alternative medicine (CAM) is used by from 22 up to 61% Western menopausal women (16). Acupuncturists rank as the second most consulted practitioners by this population (17). Acupuncture is one of the simplest, popular, and safest CAM procedures in Western countries, and has been practiced widely in China for more than 4,000 years (15). It involves insertion of one or several thin, solid, metallic needles through the skin into underlying tissues at different depths and at strategic points on the body (called “acupoints”) to produce a desired therapeutic effect (18). After insertion, the needles are usually manipulated in an up-and-down and rotating movement (i.e., manual acupuncture) or connected to a battery-operated device delivering electric microcurrent in high- or low-frequency (i.e., electroacupuncture), or a combination of both techniques (13). Acupuncture is commonly used for acute and chronic pain relief, indigestion, anxiety, insomnia, fertility and a broad range of other health conditions (19, 20). The mechanisms underlying its actions are multifaceted, including anti-inflammation, anti-oxidative stress, enhancing endogenous pain inhibition, lipolitic effects on metabolism, immunomodulator effects on the immune system, and modulation of the neurotransmitters (21, 22). Previous systematic reviews indicated that acupuncture may be effective for PMD (14) and PMI (15). However, the findings are inconclusive because of various methodological deficiencies in studies reviewed. The positive potentials of using acupuncture in the management of comorbid PMD and PMI has been summarized in a prior review by our team (23). In line with this review, the RCT presented here was designed to provide an estimate of the short-, medium-, and long- term clinical benefits that a woman with comorbid PMD and PMI might experience from undergoing a course of acupuncture treatment, relative to sham control. Given the association between perimenopausal symptoms and a complex hormonal milieu (24, 25), this trial also aimed to explore whether acupuncture might modulate hormone levels and whether this would be associated with the effects of acupuncture on PMD and PMI.

## 2. Methods

### 2.1. Trial design

This single-site, patient-assessor-blind, randomized, sham-controlled clinical trial was conducted in line with the Declaration of Helsinki. We followed the Consolidated Standards of Reporting Trials (CONSORT) (26) and Standards for Reporting Interventions in Clinical Trials of Acupuncture (STRICTA) (27) recommendations for designing and reporting of this trial. The protocol was reviewed and approved by the Human Research Ethics Committee (HREC) of the Shanghai Municipal Hospital of Traditional Chinese Medicine, Shanghai University of Traditional Chinese Medicine, Shanghai, China (No. 2020SHL-KY-42-02); and endorsed by the HREC of RMIT University, Melbourne, Australia (No. 24186). All participants were informed of the aims of this trial and randomization procedure giving them a 50% opportunity of receiving real-acupuncture (RA) or sham-acupuncture (SA), and all gave written informed consent at the time of enrollment in the trial. To increase potential participants' motivation to take part in the trial, it was explained that sham- and placebo- acupuncture has been reported to be associated with some positive outcomes [improvements in depression (28), and/or in insomnia (29)] in previous studies. All procedures including acupuncture treatment and outcome assessment were provided free of charge. This trial was registered at Chinese Clinical Trial Registry (ChiCTR) (Identifier: ChiCTR2100043054).

### 2.2. Participants

From February 2021 to December 2021, 70 participants were recruited at the Shanghai Municipal Hospital of Traditional Chinese Medicine *via* outpatient services, health promotion/education in the community, and advertisements on hospital-based posters and social media (WeChat, Tencent Holdings Limited).

Women were enrolled if they fulfilled the following criteria: (1) aged 45–55 years; (2) experiencing “perimenopause” defined according to the Stages of Reproductive Aging Workshop (STRAW) criteria (30); (3) met the International Classification of Diseases-*Ten Edition* (ICD-10) diagnostic criteria for mild or moderate depressive episode (31) and International Classification of Sleep Disorders-*Third Edition* (ICSD-3) diagnostic criteria for insomnia (32), and maintained comorbid depression and insomnia symptoms for at least 4 weeks; (4) 8 points ≤ 17 items-Hamilton Depression Rating Scale (HAM-D_17_) ≤ 23 points; (5) Pittsburgh Sleep Quality Index (PSQI) >5 points; and (6) met the diagnostic criteria for “Depression of *Liver* and Deficiency of *Kidney*” pattern in Traditional Chinese Medicine (TCM) theory (see Appendix 1).

Participants were excluded if they: (1) showed non-natural menopause transition due to premature ovarian failure, medication intake, or surgery (e.g., oophorectomy); (2) were pregnant or currently lactating; (3) depression and/or insomnia were caused by systemic diseases (e.g., stroke, Parkinson's disease, surgery, etc.); (4) were diagnosed with a severe depressive episode according to ICD-10 criteria (31) and showed suicidal ideation/attempts; (5) had a depressive disorder diagnosis prior to perimenopause; (6) comorbid with other psychiatric and mental disorders (e.g., general anxiety disorder, bipolar disorder, obsession, etc.), other depression-related disorders (e.g., recurrent depressive disorder, dysthymia, etc.), or sleep-related disorders (e.g., obstructive sleep apnea syndrome, restless legs syndrome, etc.) diagnosed according to Diagnostic and Statistical Manual of Mental Disorders-*Fifth Edition* (DSM-V) (33) and ICD-10 (31) in addition to depressive episode and insomnia; (7) had any serious physical illness, such as serious diseases of cardiovascular or hematopoietic systems, or poor liver or kidney function; (8) took medications (e.g., antidepressant, hypnotic, other psychotropic substances, Chinese herbal medicine, etc.) and/or healthcare products (e.g., melatonin, black cohosh, etc.), or received other therapies (e.g., CBT, Mindfulness-Based Exercise, etc.) that are intended to treat depression/insomnia within the last 1 month, or received HRT within the last 2 months prior to the baseline; (9) had acupuncture treatment experience in the past 6 months; (10) had an infection close to the site of the selected acupoints; (11) alcohol or drug abuse/addiction; and (12) participated in any other clinical trial within last 3 months.

### 2.3. Intervention

Participants in both groups received 17 sessions of acupuncture treatments for eight consecutive weeks (three sessions per week for the first 3 weeks, two sessions per week for the next 3 weeks, and one session per week for the final 2 weeks). All participants were provided with basic mental health and sleep hygiene education (see Appendix 2) before their first treatment session, which is the standard intervention given to all patients with climacteric-related mental illness at the trial site hospital.

#### 2.3.1. RA group

Skin around acupoints was sterilized with 75% alcohol, and then 14 standard sterilized disposable, stainless steel needles [0.25 mm in diameter and 25 mm/40 mm in length; Shanghai Jiajian Medical Instrument Co., Ltd. (Shanghai, China)] were inserted into nine acupoints, including Yintang (EX-HN3), Baihui (GV20), Guanyuan (CV4), Yinjiao (CV7) and bilateral Neiguan (PC6), Taixi (KI3), Taichong (LR3), Sanyinjiao (SP6), and Zigong (EX-CA1). The location of each acupoint adhered to the “Nomenclature and Location of Acupuncture Points [GB/T 12346-2006]” (National Standard of People's Republic of China, *2006 Version*) (34). The angle (puncture perpendicularly/obliquely/horizontally) and depth of insertion (5–15 mm) was adjusted based on the standard permissible angle and depth of insertion for each acupoint (35). After insertion, the needle was then twirled, thrust, rotated and/or lifted moderately until *De*-*qi* sensation (*Note: De-qi* refers to acupuncture-evoked specific sensations such as soreness, numbness, heaviness, and distention at the site of needle placement, and these sensations may spread to other parts of the body) was achieved. All needles were manipulated once every 10 min with intermittent stimulation during the 30-min retention period.

#### 2.3.2. SA group

Participants in the SA group received the same needling procedure and number of needles as the RA group. The main differences between the two treatment approaches were point selection and needling techniques. The acupoints selected in the RA group were those recommended in international standard guidelines [“*Indications of Acupuncture Points (GB/T 30233-2013)*” (National Standard of People's Republic of China, *2013 Version*)] (35) in relation to mental or gynecological illness, whereas the acupoints selected in the SA group were those irrelevant to either mental/sleep or gynecological disorders (see Appendix 3). Acupoints selected for the SA group included bilateral Zhouliao (LI12), Shouwuli (LI13), Tiaokou (ST38), Yangfu (GB38), Xuanzhong (GB39), Sanyangluo (TE8), and Sidu (TE9). The needles were shallowly inserted into each acupoint instead of being inserted to the required treatment depth as in routine clinical practice and no twirling, thrusting, rotating and/or lifting was carried out thus avoiding any *De*-*qi* sensation. In the SA group, participants were checked every 10 min in a similar manner to the RA group for any discomfort but without any needle manipulation.

#### 2.3.3. Other concurrent interventions

Other interventions for PMD/PMI (e.g., herbal medicine, Western medicine, melatonin, or other non-pharmacologic interventions such as Qigong, Tuina, psychotherapy, etc.) were discouraged during the treatment phase. If participants insisted on utilizing these interventions, they were asked to report the dosage and frequency of usage. This information was documented in the Case Report Form (CRF). When a participant reported AEs, this information was used to distinguish whether the AEs were caused by RA/SA or caused by any other concurrent interventions that the participant was using of their own volition.

#### 2.3.4. Rationality of acupoint selection and acupuncture dose

The selection of acupoints was on the basis of our team's review and experts' advice. Our previous systematic review indicated that the most frequently used acupoints for depression-related insomnia were EX-HN3, GV20 and SP6 (13). Given the recruited participants were within the “Depression of *Liver* and Deficiency of *Kidney*” pattern, we chose LR3 and KI3, which was *Yuan*-*Source* acupoint of *Liver Meridian of Foot-Jueyin* and *Kidney Meridian of Foot-Shaoyin*, respectively. During the trial design, we also consulted two acupuncturists with over 30-years of experience in treating insomnia and mental illness, and they agreed with the key point selection and suggested further acupoints to address other associated signs and symptoms.

Two previously published peer-reviewed articles showed that a total of 36 treatment sessions over 12 weeks (2) and a total of 18 acupuncture treatment sessions over eight weeks (36) improved PMD and PMI, respectively. For cost-effectiveness consideration and to identify the minimum effective treatment dose, we designed an 8-week treatment plan and reduced one session based on that PMI paper. We aimed to determine if such dose, which was effective for PMI, was also effective for PMI comorbid with PMD. Over the 8-week treatment period, the acupuncture dose was delivered in a gradually tapering pattern (from three sessions per week to one session per week) to mimic the withdrawal/reduction principle of antidepressant/hypnotic application. Such design is to allow the participants to better adapt to the gradual withdrawal of acupuncture treatment. Previous acupuncture trials have also used this tapering method and found it helpful in sustaining the effect of acupuncture (37, 38).

### 2.4. Outcome measures and follow-ups

Outcomes were appraised across four time-points, namely, baseline, end of the last treatment session (i.e., “post-treatment”), 8-week follow-up, and 16-week follow-up. The latter three time points were used to determine the short-, medium-, and long- term effects of acupuncture.

#### 2.4.1. Assessment of efficacy

The primary outcomes were the changes of depression and insomnia severity scores from baseline to 16-week follow-up, measured by the HAM-D_17_ (39) and PSQI (40). Secondary outcomes included (1) Meno-D (41), a scale designed to assess PMD symptoms; (2) Insomnia Severity Index (ISI) (42), an instrument to assess severity of insomnia; (3) Kupperman Index (KI) (43), a questionnaire used to evaluate climacteric symptoms; (4) Menopause-Specific Quality-of-Life (MenQoL) (44), an inventory used to rate health-related quality of life (QoL) in the menopausal period; (5) Hamilton Anxiety Rating Scale (HAM-A) (45), a measure of anxiety; (6) serum sex hormone levels [follicle-stimulating hormone (FSH); estradiol (E_2_); luteinizing hormone (LH)]; (7) Sleep Hygiene Behavior Checklist (SHBC), a checklist developed by our team. The checklist consists of 10 bad sleep hygiene behaviors/habits, which may directly affect participants' sleep quality and even the effects of acupuncture treatment (see Appendix 4); (8) Social Readjustment Rating Scale (SRRS), a scale to quantify the impacts of life events and stress on mood and sleep (46); and (9) Simplified Coping Style Questionnaire (SCSQ), a questionnaire to evaluate ability to cope with different stressful life events (47). The schedule for enrolment and the assessment time-points of each outcome measure are displayed in Appendix 5.

#### 2.4.2. Assessment of safety

Any AEs, such as unfavorable or unintended signs, symptoms, or diseases (derived from RA/SA or co-interventions) whether self-reported or identified by acupuncturist (H-X), as well as the solution for AEs and the final prognosis (AEs was relieved fast/AEs persist/AEs got worse) were followed up and recorded in detail in the CRF.

#### 2.4.3. Assessment of credibility

Blinding is a critical methodological feature of RCTs to minimize bias and maximize the validity of the results (48). At the post-treatment, the Acupuncture Perception (Credibility) Scale (APS) (49) with both the James' blinding index (50) and Bang's blinding index (51) were used to judge if the participants could distinguish whether treatment received was RA or SA.

#### 2.4.4. Assessment of participants' expectation, experience, and satisfaction of acupuncture

At baseline and the end of ninth treatment session, the Acupuncture Expectancy Scale (AES) (52) was adopted to allow participants to quantify their expectations of acupuncture on the symptoms. The Massachusetts General Hospital Acupuncture Sensation Scale (MASS) (53) was assessed after completing the ninth treatment session to quantify the intensity of various needle sensations experienced by the participants. We also employed Menopause Symptoms Treatment Satisfaction Questionnaire (MS-TSQ) (54) at the end of ninth treatment session and at post-treatment to measure participants' satisfaction levels toward acupuncture (Appendix 5).

### 2.5. Sample size

The projected sample size required was calculated on the basis of change of HAM-D_17_ and PSQI global scores. In light of the previous RCTs (55, 56) with similar designs, the difference in HAM-D_17_ scores and PSQI scores between women with PMD or PMI who received RA and those who received SA was 2.08 points and 3.73, respectively. The HAM-D_17_-based between-group difference was smaller and was therefore selected for estimation of sample size to yield a more conservative result. PASS software (Version 15.0.5, NCSS, LLC) determined no < 31 patients needed to be recruited in each group to generate statistically significant results (α = 0.05, β = 0.1; the number of patients in the two groups are equal; Two-tailed test). Hence, a total of 70 participants were required for this trial with 35 in each group, allowing for a 10% attrition rate and other risks such as non-compliance, etc.

### 2.6. Randomization, allocation concealment, and blinding

The random allocation list was generated by an independent research assistant (T-J) with no other role in this trial using SPSS software (IBM Corp., Armonk, NY, USA), and was prepared and concealed in 70 opaque, sealed, and sequentially numbered envelopes. The acupuncturist (H-X) opened the sealed envelopes once participants were at the first treatment session. If two or more participants received interventions on the same day, they were scheduled to come to the hospital at different times to avoid communication and interaction. Group allocation was kept confidential from participants, outcome assessors (FY-Z and YL-H), and the statistician (QQ-F). Only the independent research assistant (T-J) and the acupuncturist (H-X) knew the treatment allocation. The unblinding process was only executed after all the statistical analyses were completed.

### 2.7. Standardization and quality control of research

Acupuncture in either RA or SA group was delivered by the same acupuncturist (H-X) registered in the Department of Psychiatry with over 30-years acupuncture clinical experience in treating psychosomatic diseases. Furthermore, the acupuncturist was asked to minimize interactions with participants to only brief social conversation and asking about AEs to avoid non-specific treatment effects. In addition, all relevant researchers received and passed the uniform, on-site, “Clinical Research Specification Training Program” prior to the commencement of this trial. Regular onsite monitoring was carried out by trial monitoring committee to ensure the interventions were delivered as planned and data collection was carried out and documented appropriately.

### 2.8. Statistical analysis

The continuous variables were described as mean ± standard deviation (SD)/mean ± standard error (SE) or median (inter quartile range), and the categorical variables were expressed by ratios or constituent ratios. For baseline characteristics, continuous variables were examined by independent sample *t*-tests or Mann–Whitney *U* tests, and categorical variables were assessed using *Chi*-square tests, or Fisher's exact tests as appropriate. The changes of efficacy-related outcome measures (HAM-D_17_, PSQI, Meno-D, ISI, KI, MenQoL, HAM-A, and SCSQ) cross trial time-points were repeated measurement data. Paired-sample *t*-tests were adopted to address the between-group differences from baseline to each time-point; and linear mixed-effect models were adopted to detect main effect, time effect, and interaction (time^*^group) effect. We utilized paired sample *t*-tests to compare the differences of changes in serum hormone levels, SRRS, AES, and MS-TSQ between two groups. Correlational analyses were adopted between HAM-D_17_/PSQI and SRRS/SCSQ. All statistical analyses were carried out using SPSS software (IBM Corp., Armonk, NY, USA).

The James' (50) and the Bang's blinding indices (51) were analyzed using the Stata software (Version 17.0, StataCorp. LLC, College Station, TX, USA) with the blinding module.

All enrolled cases were analyzed by Intention-To-Treat (ITT) analysis, and the missing values were imputed using multiple imputation approach. We first used SPSS software to replace missing data with multiple (*n* = 5) imputations [predictive mean matching (PMM) model]; and then, the resulting *F* statistics were pooled using “miceadds:: micombine. F” in *R* software (Version 4.2.0, *R* Core Team), referring to the methodology of a previous RCT (57). However, as suggested by a previous paper regarding statistical methodology, when the proportion of missing values was more than 40%, Per-Protocol (PP) analysis should be used (58). Therefore, for those outcome measures which were assessed ≥3 time-points but the missing values were more than 40% in any time-point after baseline, we adopted both ITT analysis (using dataset obtained by multiple imputation) and PP analysis (using dataset including only remaining data after removal of missing values) to ensure the rigor of the results.

All tests in this study were two-tailed and the significance level was set to *p* < 0.05. All confidence intervals (*Cl*) were two-sided 95% intervals between the two groups.

## 3. Results

### 3.1. Participants characteristics

**Figure 1:**
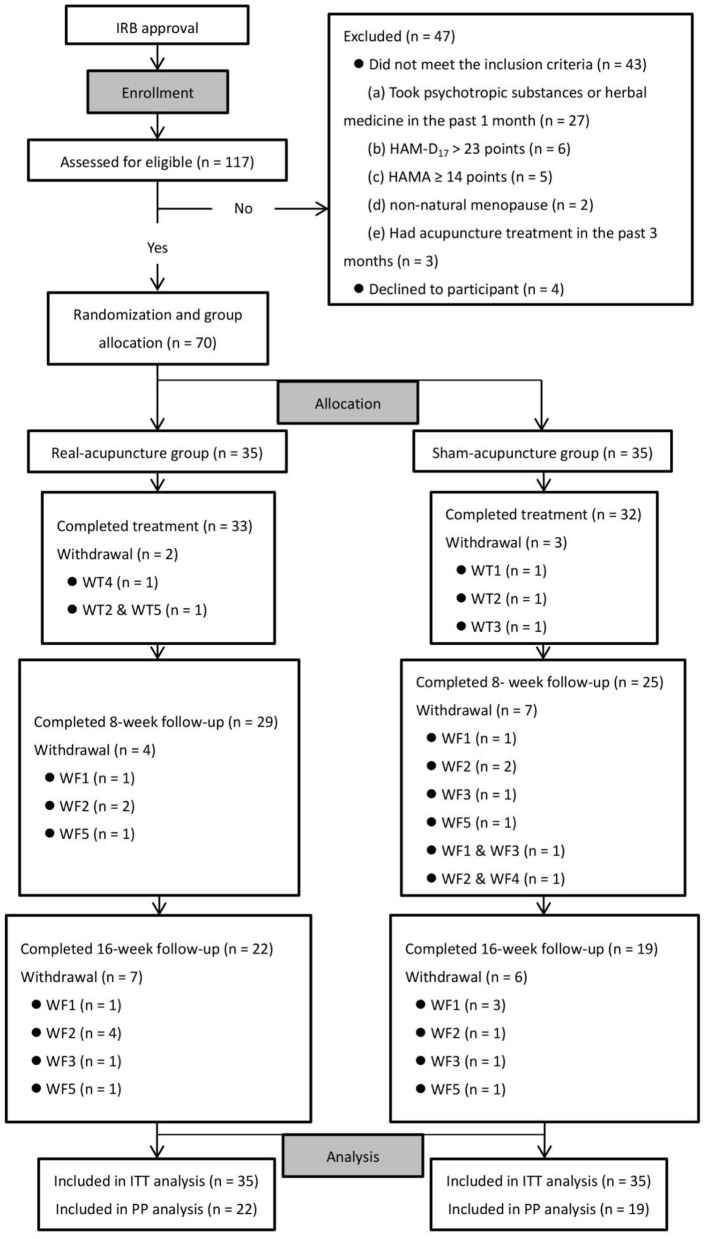
Trial flow diagram. Reasons for WT (WT1, unable to arrange time to receive treatment due to changes in work schedule; WT2, reluctant to receive further treatment because she feels it does not work as expected; WT3, consider that the treatment has achieved the desired effect and no further treatment is required; WT4, unable to continue this trial because she is under treatment for other serious medical conditions; WT5, reluctant to receive further treatment as the COVID-19 outbreak has become severe and she is worried that coming to the hospital would increase her risk of contracting COVID-19); reasons for WF [WF1, reluctant to receive follow-up and assessment as she feels the procedures are time-consuming and very cumbersome (not associated with the COVID-19 outbreak); WF2, reluctant to receive follow-up and assessment as she feels the procedures are time-consuming and very cumbersome (associated with the COVID-19 outbreak); WF3, reluctant to receive further follow-up because she feels the treatment does not work as expected; WF4, consider that the treatment has achieved the desired effect and no further follow-up is required; WF5, lost contact without being informed the specific reasons]. WT, withdrawal at treatment phase; WF, withdrawal at follow-up phase; ITT, Intention-To-Treat analysis; PP, Per-Protocol analysis.

A total of 117 participants were screened for eligibility and 47 were excluded. The remaining 70 participants were randomly assigned to receive RA (*n* = 35) or SA (*n* = 35). Of these, 65 (92.9%) completed all 17-week treatment sessions, 54 (77.1%) completed the 8-week follow-up visit, and 41 (58.6%) completed the 16-week follow-up. Reasons for withdrawal are displayed in the flow diagram (Figure 1), and the numbers and reasons are similar between the two groups.

Tables 1, 2 demonstrate the socio-demographic and clinical characteristics of the study participants. Sixty-six (94.3%) participants were married or cohabiting, and 64 (91.4%) had a secondary school diploma/degree or higher. There were no alcohol users among the participants, whereas some had a daily coffee/tea drinking habit. The mean age of participants was 48.9 years old. The demographic factors and baseline outcome assessments were comparable between the RA and SA groups (all *p* > 0.05).

**Table 1:** Socio-demographic characteristics of participants (participants = 70).

**Characteristics**	**RA (*n* = 35)**	**SA (*n* = 35)**	***p*-value**
**Age, years (Mean** ±**SD)**	48.94 ± 2.25	48.80 ± 2.07	0.78
**Race**, ***n*** **(%)**			
Han	34 (97.14)	35 (100)	1.00
Others	1 (2.86)	0 (0.00)	
**Educational level**, ***n*** **(%)**			0.52
Primary education or less	3 (8.57)	3 (8.57)	
Secondary education	18 (51.43)	13 (37.14)	
Tertiary education	14 (40.00)	19 (54.29)	
**Marital status**, ***n*** **(%)**			0.61
Married/cohabiting	32 (91.43)	34 (97.14)	
Never married	0 (0.00)	0 (0.00)	
Divorced	2 (5.71)	1 (2.86)	
Widowed	1 (2.86)	0 (0.00)	
**Occupational status**, ***n*** **(%)**			0.84
Employed	24 (68.57)	26 (74.29)	
Unemployed/house worker	2 (5.71)	1 (2.86)	
Pensioner	9 (25.71)	8 (22.86)	
**Alcohol use**, ***n*** **(%)**			N/A
Yes	0 (0.00)	0 (0.00)	
No	35 (100)	35 (100)	
**Coffee use**, ***n*** **(%)**			0.53
Yes	7 (20.00)	5 (14.29)	
No	28 (80.00)	30 (85.71)	
**Tea use**, ***n*** **(%)**			1.00
Yes	32 (91.43)	33 (94.29)	
No	3 (8.57)	2 (5.71)	

Data are presented as either Mean ± Standard Deviation or number (%).

SD, Standard Deviation; RA, real-acupuncture; SA, sham-acupuncture; N/A, not applicable.

**Table 2:** Clinical characteristics of participants at baseline (participants = 70).

**Outcomes**	**RA (*n* = 35),** **Mean ±SE**	**SA (*n* = 35),** **Mean ±SE**	**Between-group effect size (95% CI)**	***p*-value**
**HAM-D**_17_	13.23 ± 0.52	12.43 ± 0.46	0.80 (−0.58 to 2.18)	0.25
**PSQI**	11.00 ± 0.34	10.23 ± 0.29	0.77 (−0.12 to 1.66)	0.09
**Meno-D**	23.46 ± 0.59	22.40 ± 0.48	1.06 (−0.46 to 2.57)	0.17
**ISI**	13.86 ± 0.37	13.40 ± 0.33	0.46 (−0.54 to 1.45)	0.36
**KI**	16.29 ± 0.56	15.94 ± 0.50	0.34 (−1.15 to 1.84)	0.65
**MenQoL**				
Vasomotor	3.51 ± 0.10	3.49 ± 0.09	0.03 (−0.24 to 0.30)	0.84
Psychosocial	3.05 ± 0.09	2.93 ± 0.07	0.12 (−0.11 to 0.34)	0.31
Physical	3.07 ± 0.04	3.04 ± 0.04	0.03 (−0.09 to 0.14)	0.64
Sexual	3.25 ± 0.12	3.20 ± 0.12	0.05 (−0.29 to 0.39)	0.78
**HAM–A**	7.11 ± 0.29	6.66 ± 0.25	0.46 (−0.30 to 1.22)	0.24
**Reproductive hormone levels**				
FSH	37.97 ± 3.40	39.60 ± 3.39	−1.63 (−11.22 to 7.95)	0.74
E_2_	175.54 ± 16.88	190.26 ± 16.80	−14.71 (−62.24 to 32.81)	0.54
LH	18.78 ± 1.57	19.63 ± 1.42	−0.85 (−5.08 to 3.38)	0.69
**SHBC**	5.46 ± 0.21	5.40 ± 0.21	0.06 (−0.54 to 0.65)	0.85
**SCSQ**				
Positive coping	17.09 ± 0.35	17.60 ± 0.26	−0.51 (−1.39 to 0.36)	0.24
Negative coping	10.57 ± 0.25	9.89 ± 0.26	0.69 (−0.03 to 1.40)	0.06
**SRRS**	104.17 ± 2.62	100.34 ± 2.63	3.83 (−3.57 to 11.23)	0.31
**AES**	9.83 ± 0.37	9.23 ± 0.33	0.60 (−0.38 to 1.58)	0.23

Data are presented as Mean ± Standard Error.

SE, standard error; RA, real-acupuncture; SA, sham-acupuncture; CI, confidence interval; HAM-D_17_, 17 items-Hamilton Depression Rating Scale; PSQI, Pittsburgh Sleep Quality Index; ISI, Insomnia Severity Index; KI, Kupperman Index; MenQoL, Menopause-specific Quality of Life; HAM-A, Hamilton Anxiety Scale; SHBC, Sleep Hygiene Behavior Checklist; SCSQ, Simplified Coping Style Questionnaire; SRRS, Social Readjustment Rating Scale; AES, Acupuncture Expectancy Scale; FSH, follicle-stimulating hormone; E_2_, estradiol; LH, luteinizing hormone.

### 3.2. Efficacy

Twenty-nine participants (41.4%) withdrew from this trial at the 16-week follow-up. We imputed missing data per description in the methods. For those outcomes involving assessments at follow-ups, we performed both *t*-tests and linear mixed-effect models analysis using the ITT dataset (data of all 70 participants) and PP dataset (data of 41 participants who complete all assessments), respectively. The results derived from both datasets are displayed, compared and analyzed as follows. For the outcomes only assessed at baseline and post-treatment (withdrawal participants = 5, 7.1%), *t*-tests were used.

#### 3.2.1. Mood, sleep, and perimenopausal symptoms

As shown in the ITT analysis, in comparison with baseline, both groups reported significantly lower HAM-D_17_ scores at post-treatment and 8-week follow-up, but the mean differences between the two groups at each time point were not statistically significant [−1.16 (95% *CI*, −2.87 to 0.56), *p* = 0.19 at post-treatment; −0.30 (95% *CI*, −2.05 to 1.46), *p* = 0.74 at 8-week follow-up]. The similar trends were detected in the changes of Meno-D scores (Table 3). PSQI scores in both groups reduced at post-treatment and at 8-week follow-up, with RA group having a greater reduction. The ITT analysis showed statistically significantly between-group differences [−1.99 (95% *CI*, −3.02 to −0.97), *p* < 0.01 at post-treatment, and −2.34 (95% *CI*, −3.76 to −0.92), *p* < 0.01 at 8-week follow-up]. The changes of ISI scores showed a similar trend. The results of HAM-D_17_, PSQI, Meno-D, and ISI derived from the PP analysis were consistent (Table 4). Whilst the ITT analysis showed a significant time × group interaction for ISI (*p* = 0.03) but not for PSQI (*p* = 0.33), the PP analysis showed a significant time × group interaction for both ISI and PSQI (both *p* < 0.01) (Appendix 6), reflecting the sleep-promoting effect of RA was superior to that of SA over time.

**Table 3:** HAM-D_17_, PSQI, Meno-D, ISI, KI, MenQoL, HAM-A, and SHBC outcomes across trial time-points (ITT dataset, participants = 70).

**Outcome**	**RA (*****n*** = **35)**		**SA (*****n*** = **35)**		**Between-group effect size (95% CI)**	***p*-value**
	**Mean** ±**SE**	**Change from baseline**, **mean** ±**SE**	**Mean** ±**SE**	**Change from baseline**, **mean** ±**SE**		
**HAM-D**_17_						
Baseline	13.23 ± 0.52		12.43 ± 0.46			
Post-treatment	10.14 ± 0.65	−3.09 ± 0.69^a^	10.49 ± 0.40	−1.93 ± 0.54^a^	−1.16 (−2.87 to 0.56)	0.19
8-week follow-up	11.11 ± 0.47	−2.12 ± 0.56^a^	10.60 ± 0.48	−1.83 ± 0.66^a^	−0.30 (−2.05 to 1.46)	0.74
16-week follow-up	12.48 ± 0.47	−0.75 ± 0.62	12.07 ± 0.33	−0.36 ± 0.57	−0.39 (−2.04 to 1.25)	0.64
**PSQI**						
Baseline	11.00 ± 0.34		10.23 ± 0.29			
Post-treatment	8.23 ± 0.44	−2.77 ± 0.41^a^	9.45 ± 0.37	−0.78 ± 0.32^a^	−1.99 (−3.02 to −0.97)	< 0.01
8-week follow-up	8.26 ± 0.66	−2.74 ± 0.60^a^	9.84 ± 0.46	−0.39 ± 0.48	−2.34 (−3.76 to −0.92)	< 0.01
16-week follow-up	10.30 ± 0.59	−0.70 ± 0.59	10.87 ± 0.40	0.64 ± 0.50	−1.34 (−2.83 to 0.16)	0.08
**Meno-D**						
Baseline	23.46 ± 0.59		22.40 ± 0.48			
Post-treatment	19.07 ± 0.86	−4.39 ± 0.91^a^	18.83 ± 0.55	−3.57 ± 0.63^a^	−0.82 (−3.00 to 1.36)	0.46
8-week follow-up	20.42 ± 0.65	−3.03 ± 0.69^a^	20.29 ± 0.60	−2.11 ± 0.75^a^	−0.92 (−2.92 to 1.08)	0.37
16-week follow-up	22.41 ± 0.60	−1.04 ± 0.76	22.28 ± 0.46	−0.12 ± 0.65	−0.93 (−2.89 to 1.04)	0.35
**ISI**						
Baseline	13.86 ± 0.37		13.40 ± 0.33			
Post-treatment	10.77 ± 0.57	−3.08 ± 0.51^a^	12.29 ± 0.44	−1.11 ± 0.42^a^	−1.98 (−3.28 to −0.67)	< 0.01
8-week follow-up	10.83 ± 0.84	−3.03 ± 0.76^a^	13.27 ± 0.48	−0.13 ± 0.51	−2.90 (−4.72 to −1.07)	< 0.01
16-week follow-up	13.91 ± 0.64	0.05 ± 0.63	14.12 ± 0.57	0.72 ± 0.65	−0.67 (−2.51 to 1.17)	0.47
**KI**						
Baseline	16.29 ± 0.56		15.94 ± 0.50			
Post-treatment	13.79 ± 0.74	−2.50 ± 0.79^a^	13.54 ± 0.45	−2.40 ± 0.63^a^	−0.10 (−2.08 to 1.89)	0.93
8-week follow-up	14.40 ± 0.43	−1.89 ± 0.54^a^	14.00 ± 0.51	−1.94 ± 0.70^a^	0.05 (−1.67 to 1.77)	0.96
16-week follow-up	15.60 ± 0.47	−0.68 ± 0.71	15.52 ± 0.41	−0.43 ± 0.64	−0.25 (−2.09 to 1.58)	0.79
**MenQoL**						
**Vasomotor**						
Baseline	3.51 ± 0.10		3.49 ± 0.09			
Post-treatment	3.40 ± 0.12	−0.11 ± 0.13	3.41 ± 0.09	−0.08 ± 0.11	−0.03 (−0.38 to 0.31)	0.85
8-week follow-up	3.47 ± 0.10	−0.04 ± 0.13	3.44 ± 0.10	−0.05 ± 0.11	0.00 (−0.35 to 0.35)	0.99
16-week follow-up	3.53 ± 0.11	0.01 ± 0.12	3.53 ± 0.13	0.05 ± 0.14	−0.04 (−0.44 to 0.36)	0.85
**Psychosocial**						
Baseline	3.05 ± 0.09		2.93 ± 0.07			
Post-treatment	2.42 ± 0.10	−0.63 ± 0.11^a^	2.47 ± 0.07	−0.46 ± 0.08^a^	−0.17 (−0.43 to 0.09)	0.20
8-week follow-up	2.60 ± 0.09	−0.44 ± 0.11^a^	2.57 ± 0.11	−0.36 ± 0.14^a^	−0.08 (−0.46 to 0.30)	0.67
16-week follow-up	2.85 ± 0.09	−0.19 ± 0.11	2.75 ± 0.11	−0.18 ± 0.14	−0.01 (−0.40 to 0.38)	0.95
**Physical**						
Baseline	3.07 ± 0.04		3.04 ± 0.04			
Post-treatment	2.89 ± 0.06	−0.18 ± 0.07^a^	2.84 ± 0.04	−0.20 ± 0.06^a^	0.02 (−0.15 to 0.19)	0.81
8-week follow-up	2.98 ± 0.07	−0.09 ± 0.07	2.91 ± 0.07	−0.13 ± 0.08	0.04 (−0.21 to 0.29)	0.76
16-week follow-up	3.02 ± 0.09	−0.04 ± 0.09	2.99 ± 0.07	−0.06 ± 0.07	0.01 (−0.23 to 0.25)	0.93
**Sexual**						
Baseline	3.25 ± 0.12		3.20 ± 0.12			
Post-treatment	3.23 ± 0.12	−0.02 ± 0.06	3.26 ± 0.11	0.06 ± 0.08	−0.08 (−0.27 to 0.12)	0.45
8-week follow-up	3.27 ± 0.14	0.02 ± 0.12	3.28 ± 0.13	0.08 ± 0.12	−0.06 (−0.43 to 0.32)	0.75
16-week follow-up	3.29 ± 0.14	0.05 ± 0.13	3.25 ± 0.11	0.05 ± 0.14	−0.01 (−0.37 to 0.36)	0.98
**HAM-A**						
Baseline	7.11 ± 0.29		6.66 ± 0.25			
Post-treatment	5.63 ± 0.32	−1.49 ± 0.34^a^	6.34 ± 0.26	−0.32 ± 0.29	−1.17 (−2.04 to −0.30)	0.01
8-week follow-up	6.37 ± 0.47	−0.75 ± 0.48	7.51 ± 0.39	0.85 ± 0.45	−1.60 (−2.86 to −0.34)	0.01
16-week follow-up	7.04 ± 0.40	−0.07 ± 0.45	7.37 ± 0.44	0.71 ± 0.52	−0.78 (−2.23 to 0.67)	0.29
**SHBC**						
Baseline	5.46 ± 0.21		5.40 ± 0.21			
Post-treatment	3.90 ± 0.23	−1.56 ± 0.24^a^	3.93 ± 0.18	−1.47 ± 0.24^a^	−0.09 (−0.77 to 0.59)	0.80
16-week follow-up	4.43 ± 0.22	−1.02 ± 0.29^a^	4.37 ± 0.17	−1.03 ± 0.25^a^	0.01 (−0.75 to 0.76)	0.98

Data are presented as Mean ± Standard Error.

^*a*^p < 0.05, within-group effect size show statistically significant difference.

SE, standard error; RA, real-acupuncture; SA, sham-acupuncture; CI, confidence interval; HAM-D_17_, 17 items-Hamilton Depression Rating Scale; PSQI, Pittsburgh Sleep Quality Index; ISI, Insomnia Severity Index; KI, Kupperman Index; MenQoL, Menopause-specific Quality of Life; HAM-A, Hamilton Anxiety Scale; SHBC, Sleep Hygiene Behavior Checklist.

**Table 4:** HAM-D_17_, PSQI, Meno-D, ISI, KI, MenQoL, HAM-A, and SHBC outcomes across trial time-points (PP dataset, participants = 41).

**Outcome**	**RA (*****n*** = **22)**		**SA (*****n*** = **19)**		**Between-group effect size (95% CI)**	***p*-value**
	**Mean** ±**SE**	**Change from baseline**, **mean** ±**SE**	**Mean** ±**SE**	**Change from baseline**, **mean** ±**SE**		
**HAM-D**_17_						
Baseline	12.73 ± 0.71		12.89 ± 0.63			
Post-treatment	9.82 ± 0.85	−2.91 ± 0.97^a^	10.79 ± 0.41	−2.11 ± 0.58^a^	−0.80 (−3.18 to 1.57)	0.50
8-week follow-up	10.41 ± 0.56	−2.32 ± 0.81^a^	10.53 ± 0.56	−2.37 ± 0.74^a^	0.05 (−2.20 to 2.30)	0.96
16-week follow-up	12.50 ± 0.64	−0.23 ± 0.81	11.95 ± 0.41	−0.95 ± 0.76	0.72 (−1.55 to 2.99)	0.52
**PSQI**						
Baseline	10.86 ± 0.45		10.47 ± 0.39			
Post-treatment	7.91 ± 0.58	−2.95 ± 0.56^a^	9.58 ± 0.46	−0.89 ± 0.31	−2.06 (−3.42 to −0.70)	< 0.01
8-week follow-up	7.09 ± 0.75	−3.77 ± 0.74^a^	10.37 ± 0.51	−0.11 ± 0.44	−3.67 (−5.48 to −1.85)	< 0.01
16-week follow-up	10.05 ± 0.74	−0.82 ± 0.70	11.05 ± 0.54	0.58 ± 0.66	−1.40 (−3.36 to 0.57)	0.16
**Meno-D**						
Baseline	22.73 ± 0.77		23.00 ± 0.65			
Post-treatment	18.77 ± 1.19	−3.95 ± 1.31^a^	19.11 ± 0.57	−3.89 ± 0.64^a^	−0.06 (−3.16 to 3.04)	0.97
8-week follow-up	19.45 ± 0.76	−3.27 ± 0.97^a^	20.26 ± 0.76	−2.74 ± 0.83^a^	−0.54 (−3.16 to 2.09)	0.68
16-week follow-up	22.32 ± 0.88	−0.41 ± 1.01	22.32 ± 0.69	−0.68 ± 0.97	0.28 (−2.59 to 3.14)	0.85
**ISI**						
Baseline	13.77 ± 0.47		13.58 ± 0.43			
Post-treatment	10.45 ± 0.79	−3.32 ± 0.70^a^	12.53 ± 0.55	−1.05 ± 0.40	−2.27 (−3.97 to −0.56)	0.01
8-week follow-up	9.27 ± 0.99	−4.50 ± 0.96^a^	13.74 ± 0.61	0.16 ± 0.53	−4.66 (−6.97 to −2.35)	< 0.01
16-week follow-up	13.68 ± 0.98	−0.09 ± 0.88	14.53 ± 0.70	0.95 ± 0.79	−1.04 (−3.46 to 1.38)	0.39
**KI**						
Baseline	15.68 ± 0.76		16.53 ± 0.65			
Post-treatment	13.45 ± 1.01	−2.23 ± 1.12	13.95 ± 0.51	−2.58 ± 0.71^a^	0.35 (−2.44 to 3.14)	0.80
8-week follow-up	13.91 ± 0.54	−1.77 ± 0.77	13.89 ± 0.60	−2.63 ± 0.75^a^	0.86 (−1.33 to 3.04)	0.43
16-week follow-up	15.82 ± 0.66	0.14 ± 0.95	15.37 ± 0.41	−1.16 ± 0.70	1.29 (−1.16 to 3.75)	0.29
**MenQoL**						
**Vasomotor**						
Baseline	3.35 ± 0.13		3.60 ± 0.12			
Post-treatment	3.32 ± 0.15	−0.03 ± 0.18	3.51 ± 0.12	−0.09 ± 0.10	0.06 (−0.38 to 0.50)	0.79
8-week follow-up	3.42 ± 0.11	0.08 ± 0.17	3.49 ± 0.12	−0.10 ± 0.11	0.18 (−0.25 to 0.61)	0.40
16-week follow-up	3.53 ± 0.11	0.18 ± 0.11	3.47 ± 0.12	−0.12 ± 0.08	0.31 (0.03 to 0.58)	0.03
**Psychosocial**						
Baseline	2.98 ± 0.11		3.00 ± 0.10			
Post-treatment	2.37 ± 0.12	−0.61 ± 0.15^a^	2.54 ± 0.06	−0.46 ± 0.09^a^	−0.15 (−0.50 to 0.21)	0.41
8-week follow-up	2.55 ± 0.09	−0.42 ± 0.14^a^	2.53 ± 0.08	−0.47 ± 0.12^a^	0.05 (−0.33 to 0.43)	0.79
16-week follow-up	2.85 ± 0.09	−0.13 ± 0.12	2.77 ± 0.06	−0.23 ± 0.12	0.10 (−0.25 to 0.45)	0.55
**Physical**						
Baseline	3.01 ± 0.06		3.09 ± 0.05			
Post-treatment	2.87 ± 0.08	−0.14 ± 0.09	2.88 ± 0.04	−0.21 ± 0.07^a^	0.07 (−0.16 to 0.29)	0.57
8-week follow-up	2.94 ± 0.04	−0.08 ± 0.06	2.92 ± 0.06	−0.16 ± 0.08	0.09 (−0.11 to 0.28)	0.38
16-week follow-up	3.03 ± 0.07	0.02 ± 0.08	3.01 ± 0.04	−0.07 ± 0.06	0.09 (−0.12 to 0.30)	0.40
**Sexual**						
Baseline	3.11 ± 0.16		3.40 ± 0.15			
Post-treatment	3.12 ± 0.16	0.02 ± 0.04	3.30 ± 0.14	−0.10 ± 0.04	0.12 (0.00 to 0.24)	0.05
8-week follow-up	3.23 ± 0.14	0.12 ± 0.06	3.37 ± 0.14	−0.03 ± 0.05	0.16 (0.00 to 0.31)	0.05
16-week follow-up	3.27 ± 0.14	0.17 ± 0.05	3.23 ± 0.15	−0.18 ± 0.09	0.34 (0.14 to 0.55)	< 0.01
**HAM-A**						
Baseline	7.00 ± 0.39		6.89 ± 0.30			
Post-treatment	5.50 ± 0.45	−1.50 ± 0.47^a^	6.42 ± 0.34	−0.47 ± 0.28	−1.03 (−2.18 to 0.12)	0.08
8-week follow-up	5.41 ± 0.46	−1.59 ± 0.54^a^	7.47 ± 0.42	0.58 ± 0.41	−2.17 (−3.58 to −0.76)	< 0.01
16-week follow-up	7.00 ± 0.57	0.00 ± 0.61	7.47 ± 0.53	0.58 ± 0.66	−0.58 (−2.40 to 1.24)	0.52
**SHBC**						
Baseline	5.55 ± 0.24		5.68 ± 0.30			
Post-treatment	3.77 ± 0.28	−1.77 ± 0.30^a^	4.26 ± 0.17	−1.42 ± 0.27^a^	−0.35 (−1.18 to 0.48)	0.40
16-week follow-up	4.45 ± 0.29	−1.09 ± 0.36^a^	4.37 ± 0.22	−1.32 ± 0.32^a^	0.22 (−0.77 to 1.22)	0.65

Data are presented as Mean ± Standard Error.

^*a*^p < 0.05, within-group effect size show statistically significant difference.

SE, standard error; RA, real-acupuncture; SA, sham-acupuncture; CI, confidence interval; HAM-D_17_, 17 items-Hamilton Depression Rating Scale; PSQI, Pittsburgh Sleep Quality Index; ISI, Insomnia Severity Index; KI, Kupperman Index; MenQoL, Menopause-specific Quality of Life; HAM-A, Hamilton Anxiety Scale; SHBC, Sleep Hygiene Behavior Checklist.

The ITT analysis showed that the RA group reported a greater reduction in HAM-A scores when compared with the SA group at 8-week follow-up [−1.60 (95% *CI*, −2.86 to −0.34), *p* = 0.01] (Table 3). The PP analysis showed similar results (Table 4). The linear mixed-effects models did not show a significant time × group interaction for HAM-A, according to the ITT analysis. However, the PP analysis showed an opposite result (*p* = 0.04), as at 8-week follow-up, HAM-A scores continued to decline in the RA group, but increased in the SA group (Appendix 6).

In the sexual dimension of MenQoL, contrary to what we anticipated, whilst the ITT analysis did not show any within-group and between-group differences (Table 3), the PP analysis demonstrated that SA caused a greater reduction compared with RA did [0.34 (95% *CI*, 0.14–0.55), *p* < 0.01] at 16-week follow-up (Table 4). Also, there was a significant time × group interaction (*p* < 0.01). No significant time × group interaction was identified in other dimensions, derived from either ITT dataset or PP dataset (Appendix 6).

There was neither between-group difference nor time × group interaction in either KI or SHBC scores (Tables 3, 4, and Appendix 6).

#### 3.2.2. Ability to cope with stressful life events

We analyzed the SCSQ and SRRS scores using the ITT dataset and the PP dataset separately (Tables 5, 6). The only significant difference was the increased SRRS scores at 16-week follow-up in both groups (both *p* < 0.05 in ITT dataset), which was largely attributed to the changes in life due to the constantly deteriorative coronavirus disease 2019 (COVID-19) pandemic in Shanghai from late 2021 to the June 2022, in accordance with participants' self-reports. The linear mixed-effects models did not show a significant time × group interaction in either positive coping or negative coping dimension of SCSQ, based on either the ITT or PP analyses (Appendix 6).

**Table 5:** SCSQ and SRRS outcomes across trial time-points (ITT dataset, participants = 70).

**Outcome**	**RA (*****n*** = **35)**		**SA (*****n*** = **35)**		**Between-group effect size (95% CI)**	***p*-value**
	**Mean** ±**SE**	**Change from baseline**, **mean** ±**SE**	**Mean** ±**SE**	**Change from baseline**, **mean** ±**SE**		
**SCSQ**						
**Positive coping**						
Baseline	17.09 ± 0.35		17.60 ± 0.26			
Post-treatment	17.39 ± 0.36	0.31 ± 0.29	17.66 ± 0.27	0.06 ± 0.25	0.25 (−0.50 to 1.00)	0.51
16-week follow-up	17.29 ± 0.42	0.20 ± 0.42	17.36 ± 0.26	−0.24 ± 0.29	0.43 (−0.63 to 1.50)	0.41
**Negative coping**						
Baseline	10.57 ± 0.25		9.89 ± 0.26			
Post-treatment	10.42 ± 0.32	−0.15 ± 0.26	9.85 ± 0.24	−0.03 ± 0.20	−0.16 (−0.94 to 0.63)	0.69
16-week follow-up	10.78 ± 0.28	0.21 ± 0.28	10.25 ± 0.30	0.37 ± 0.26	−0.12 (−0.76 to 0.51)	0.70
**SRRS**						
Baseline	104.17 ± 2.62		100.34 ± 2.63			
16-week follow-up	108.85 ± 2.33	4.68 ± 1.51^a^	106.58 ± 2.30	6.23 ± 1.89^a^	−1.55 (−6.33 to 3.22)	0.52

Data are presented as Mean ± Standard Error.

^*a*^p < 0.05, within-group effect size show statistically significant difference.

SE, standard error; RA, real-acupuncture; SA, sham-acupuncture; CI, confidence interval; SCSQ, Simplified Coping Style Questionnaire; SRRS, Social Readjustment Rating Scale.

**Table 6:** SCSQ and SRRS outcomes across trial time-points (PP dataset, participants = 41).

**Outcome**	**RA (*****n*** = **22)**		**SA (*****n*** = **19)**		**Between-group effect size (95% CI)**	***p*-value**
	**Mean** ±**SE**	**Change from baseline**, **mean** ±**SE**	**Mean** ±**SE**	**Change from baseline**, **mean** ±**SE**		
**SCSQ**						
**Positive coping**						
Baseline	17.45 ± 0.44		17.47 ± 0.35			
Post-treatment	17.68 ± 0.44	0.23 ± 0.36	17.63 ± 0.29	0.16 ± 0.24	0.07 (−0.84 to 0.98)	0.88
16-week follow-up	17.18 ± 0.44	−0.27 ± 0.27	17.37 ± 0.34	−0.11 ± 0.21	−0.17 (−0.89 to 0.55)	0.64
**Negative coping**						
Baseline	10.50 ± 0.32		10.00 ± 0.37			
Post-treatment	10.36 ± 0.39	−0.14 ± 0.33	9.79 ± 0.34	−0.21 ± 0.25	0.07 (−0.79 to 0.93)	0.86
16-week follow-up	10.91 ± 0.31	0.41 ± 0.24	10.11 ± 0.42	0.11 ± 0.23	0.30 (−0.38 to 0.99)	0.37
**SRRS**						
Baseline	107.14 ± 3.72		102.26 ± 4.15			
16-week follow-up	108.86 ± 3.62	1.73 ± 1.19	106.37 ± 4.12	4.11 ± 1.87	−2.38 (−6.75 to 1.99)	0.28

Data are presented as Mean ± Standard Error.

SE, standard error; RA, real-acupuncture; SA, sham-acupuncture; CI, confidence interval; SCSQ, Simplified Coping Style Questionnaire; SRRS, Social Readjustment Rating Scale.

We explored the correlation between HAM-D_17_ and PSQI with SRRS and SCSQ as we considered depression and insomnia might be related to social adjustment or coping. Table 7 showed HAM-D_17_ and PSQI scores were positively correlated with the negative coping dimension of SCSQ, and negatively correlated with the positive coping dimension of SCSQ, reflecting those who were more depressed or with poorer sleep were more likely to adopt negative coping strategies. There was no significant correlation between SRRS and HAM-D_17_/PSQI scores.

**Table 7:** Correlational analyses between HAM-D_17_/PSQI and SRRS/SCSQ (ITT dataset, participants = 70).

**Outcomes**	**SRRS**		**SCSQ**			
			**Positive coping**		**Negative coping**	
	***r*** **-value**	***p*** **-value**	***r*** **-value**	***p*** **-value**	***r*** **-value**	***p*** **-value**
**HAMD**	0.09	0.44	−0.87	< 0.01	0.73	< 0.01
**PSQI**	0.18	0.14	−0.79	< 0.01	0.66	< 0.01

HAM-D_17_, 17 items-Hamilton Depression Rating Scale; PSQI, Pittsburgh Sleep Quality Index; SCSQ, Simplified Coping Style Questionnaire; SRRS, Social Readjustment Rating Scale.

#### 3.2.3. Reproductive hormone levels

Analysis of PP dataset was unnecessary given only five participants did not provide data for post-treatment assessment on serum reproductive hormone levels. Changes in hormone levels between baseline and post-treatment are presented in Table 8. After treatment, both groups showed a trend of an increase in FSH and LH levels and a trend of decrease in E_2_ levels. However, there was no significant between-group difference in all three hormone levels (all *p* > 0.05).

**Table 8:** Serum reproductive hormone levels across trial time-points (ITT dataset, participants = 70).

**Outcome**	**RA (*****n*** = **35)**		**SA (*****n*** = **35)**		**Between-group effect size (95% CI)**	***p*-value**
	**Mean** ±**SE**	**Change from baseline**, **mean** ±**SE**	**Mean** ±**SE**	**Change from baseline**, **mean** ±**SE**		
**FSH (mIU/mL)**						
Baseline	37.97 ± 3.40		39.60 ± 3.39			
Post-treatment	39.97 ± 3.40	2.00 ± 1.56	42.67 ± 3.89	3.07 ± 2.29	−1.07 (−6.50 to 4.35)	0.70
**E**_2_ **(pmol/L)**						
Baseline	175.54 ± 16.88		190.26 ± 16.80			
Post-treatment	154.98 ± 14.03	−20.57 ± 11.89	160.78 ± 12.91	−29.48 ± 10.73^a^	8.91 (−22.48 to 40.30)	0.58
**LH (mIU/mL)**						
Baseline	18.78 ± 1.57		19.63 ± 1.42			
Post-treatment	20.39 ± 1.49	1.60 ± 0.76^a^	20.17 ± 1.58	0.53 ± 1.08	1.07 (−1.53 to 3.68)	0.42

Data are presented as Mean ± Standard Error.

^*a*^p < 0.05, within-group effect size show statistically significant difference.

SE, standard error; RA, real-acupuncture; SA, sham-acupuncture; CI, confidence interval; FSH, follicle-stimulating hormone; E_2_, estradiol; LH, luteinizing hormone.

### 3.3. Participants' expectation, experience, and satisfaction of acupuncture

Each participant was asked to rate their experience of needling sensations using the MASS scale. Sixty-six participants (34 in RA group; 32 in SA group) who completed at least nine treatment sessions completed this appraisal (Table 9). Thirty-three (97%) participants in the RA group experienced mild to moderate soreness and 14 (41%) experienced fullness/distension. Whereas, in the SA group, sharp pain was the main sensation experienced by 22 (69%) participants; and 10 participants (31%) also experienced aching. We further analyzed the intensity of each sensation using the Mann–Whitney *U* test (Table 10). There were significant differences in soreness, fullness/distension, numbness, and sharp pain (all *p* < 0.01), with the RA group more likely reporting a higher degree of soreness, fullness/distension, and numbness whereas the SA group reporting a higher degree of sharp pain.

**Table 9:** Proportion of each participants-reported needle sensation recorded by MASS (participants = 66).

**MASS**	**Soreness**	**Aching**	**Deep** **pressure**	**Heaviness**	**Fullness/** **distension**	**Tingling**	**Numbness**	**Sharp pain**	**Dull pain**	**Warmth**	**Cold**	**Throbbing**	**Other**
RA (*n* = 34)	33 (97.06)	5 (14.70)	0	0	14 (41.18)	0	7 (20.59)	10 (29.41)	0	0	0	0	0
SA (*n* = 32)	3 (9.38)	10 (31.25)	0	0	0	0	0	22 (68.75)	0	0	0	0	0

Data are presented as number (%).

RA, real-acupuncture; SA, sham-acupuncture; MASS, Massachusetts General Hospital Acupuncture Sensation Scale.

**Table 10:** Degree of each participants-reported needle sensation recorded by MASS (participants = 66).

**MASS**	**Soreness**	**Aching**	**Deep** ** pressure**	**Heaviness**	**Fullness/** **distension**	**Tingling**	**Numbness**	**Sharp pain**	**Dull pain**	**Warmth**	**Cold**	**Throbbing**	**Other**
RA (*n* = 34)	48.31	30.63	33.50	33.50	40.09	33.50	36.79	27.76	33.50	33.50	33.50	33.50	33.50
SA (*n* = 32)	17.77	36.55	33.50	33.50	26.50	33.50	30.00	39.59	33.50	33.50	33.50	33.50	33.50
U	40.50	446.50	544.00	544.00	320.00	544.00	432.00	349.00	544.00	544.00	544.00	544.00	544.00
*p*	< 0.01	0.09	1.00	1.00	< 0.01	1.00	< 0.01	< 0.01	1.00	1.00	1.00	1.00	1.00

Data are presented as Rank Mean.

RA, real-acupuncture; SA, sham-acupuncture; MASS, Massachusetts General Hospital Acupuncture Sensation Scale.

Change in participants' expectation of acupuncture from baseline to the end of end of ninth treatment session was recorded by AES scale. Both groups showed a significant downward trend in AES scores (both *p* < 0.05), although no significant between-group difference was detected [0.07 (95% *CI*, −0.61 to 0.76), *p* = 0.83] (Table 11).

**Table 11:** AES outcome across trial time-points (ITT dataset, participants = 70).

**AES**	**RA (*****n*** = **35)**		**SA (*****n*** = **35)**		**Between-group effect size (95% CI)**	***p*-value**
	**Mean** ±**SE**	**Change from baseline**, **mean** ±**SE**	**Mean** ±**SE**	**Change from baseline**, **mean** ±**SE**		
Baseline	9.83 ± 0.37		9.23 ± 0.33			
End of ninth treatment session	9.14 ± 0.40	−0.69 ± 0.20^a^	8.47 ± 0.28	−0.76 ± 0.28^a^	0.07 (−0.61 to 0.76)	0.83

Data are presented as either Mean ± Standard Error.

^*a*^p < 0.05, within-group effect size show statistically significant difference.

SE, standard error; RA, real-acupuncture; SA, sham-acupuncture; CI, confidence interval; AES, Acupuncture Expectancy Scale.

Analysis of PP dataset was unnecessary for MS-TSQ given only four and only five participants did not provide data at end of ninth treatment session and post-treatment assessments, respectively. As shown in Table 12, at post-treatment, the satisfaction scores of RA group were significantly higher than that of the SA group in three dimensions only (sleep quality, concentration, and overall) (all *p* < 0.05). The mean value of overall satisfaction scores in RA group was 3.29, between “Satisfied” and “Extremely Satisfied,” while the mean value of overall satisfaction in SA group was 2.28, between “Neutral” and “Satisfied,” according to scoring principles of MS-TSQ scale (54). As treatment progressed, the satisfaction scores of RA group increased significantly in sleep quality and overall dimensions (both *p* < 0.05), and such changes were superior to the changes in SA group [1.02 (95% *CI*, 0.61–1.43), *p* < 0.01 in sleep quality dimension; 0.86 (95% *CI*, 0.49–1.24), *p* < 0.01 in overall quality dimension].

**Table 12:** MS-TSQ outcome across trial time-points (ITT dataset, participants = 70).

**MS-TSQ**	**RA (*****n*** = **35)**		**SA (*****n*** = **35)**		**Between-group differences (95% CI)**	***p*-value**
	**Mean** ±**SE**	**Change from end of ninth treatment session**, **mean** ±**SE**	**Mean** ±**SE**	**Change from end of ninth treatment session**, **mean** ±**SE**		
**Hot flashes during day**						
End of ninth treatment session	2.00 ± 0.12		2.04 ± 0.12			
Post-treatment	2.11 ± 0.16	0.12 ± 0.14	2.17 ± 0.15	0.13 ± 0.13	−0.01 (−0.37 to 0.35)	0.96
**Hot flashes during night**						
End of ninth treatment session	2.12 ± 0.13		2.11 ± 0.12			
Post-treatment	2.20 ± 0.20	0.08 ± 0.14	2.26 ± 0.17	0.15 ± 0.14	−0.07 (−0.48 to 0.33)	0.72
**Quality of sleep**						
End of ninth treatment session	2.35 ± 0.10		2.01 ± 0.11			
Post-treatment	3.33 ± 0.19^b^	0.98 ± 0.16^a^	1.96 ± 0.18	−0.05 ± 0.14	1.02 (0.61 to 1.43)	< 0.01
**Mood and emotions**						
End of ninth treatment session	2.39 ± 0.17		2.10 ± 0.14			
Post-treatment	2.59 ± 0.24	0.21 ± 0.15	2.45 ± 0.18	0.35 ± 0.13^a^	−0.15 (−0.52 to 0.23)	0.44
**Interest in sex**						
End of ninth treatment session	2.09 ± 0.09		2.09 ± 0.08			
Post-treatment	2.24 ± 0.12	0.15 ± 0.09	2.09 ± 0.10	−0.01 ± 0.10	0.16 (−0.09 to 0.41)	0.22
**Concentration**						
End of ninth treatment session	2.04 ± 0.09		1.87 ± 0.08			
Post-treatment	2.11 ± 0.10^b^	0.07 ± 0.08	1.71 ± 0.11	−0.16 ± 0.09	0.23 (−0.02 to 0.47)	0.07
**Tolerability to side-effects**						
End of ninth treatment session	1.88 ± 0.06		1.95 ± 0.04			
Post-treatment	1.88 ± 0.06	0.00 ± 0.02	1.95 ± 0.04	−0.01 ± 0.05	0.00 (−0.09 to 0.09)	0.97
**Overall satisfaction**						
End of ninth treatment session	2.20 ± 0.10		2.06 ± 0.08			
Post-treatment	3.29 ± 0.17^b^	1.09 ± 0.15^a^	2.28 ± 0.14	0.22 ± 0.12	0.86 (0.49 to 1.24)	< 0.01

Data are presented as either Mean ± Standard Error.

^*a*^p < 0.05, within-group effect size show statistically significant difference in difference value from end of ninth treatment session to post-treatment.

^*b*^p < 0.05, between-group effect size show statistically significant difference between RA and SA at post-treatment.

SE, standard error; RA, real-acupuncture; SA, sham-acupuncture; CI, confidence interval; MS-TSQ, Menopause Symptoms Treatment Satisfaction Questionnaire.

### 3.4. Adverse events

In the RA group, four (11.43%) participants reported pain at needling sites, and two of them also reported subcutaneous hematoma around needling sites. In SA group, two (5.71%) participants reported dizziness, and one of them also reported pain at needling sites; another (2.86%) reported numbness in the arms. All AEs were considered mild by participants. Moreover, according to participants' self-reports, these AEs usually appeared after the needles were removed and gradually disappeared over the following 24–72 h. None of the participants withdrew due to AEs or required medical attention.

### 3.5. Success of blinding and credibility assessment

Sixty-five participants who received all treatment sessions completed the APS appraisal, in which participants were asked to make a guess of their group allocations as well as the basis for their guess. As exhibited in Table 13, a majority of participants in either group judged the type of acupuncture treatment they received based on its clinical effectiveness.

**Table 13:** Results of blinding assessed by James' blinding index and Bang's blinding index (participants = 66).

**Group allocation**	**Basis for judgement**, ***N*** **(%)**					**Guess on the treatment received**, ***N*** **(%)**			**James' blinding index (95% CI)**	**Bang's blinding index (95% CI)**
	**A**	**B**	**C**	**D**	**E**	**RA**	**SA**	**Uncertain**		
RA (*n* = 33)	0 (0)	0 (0)	1 (3.03)	32 (96.97)	0 (0)	22 (66.67)^a^	10 (30.30)	1 (3.03)	0.67 (0.59 to 0.75)	0.38 (0.23 to 0.53)
SA (*n* = 32)	6 (18.75)	0 (0)	3 (9.38)	23 (71.88)	0 (0)	12 (37.50)	14 (43.75)^a^	6 (18.75)		−0.14 (−0.33 to 0.05)

Basis for judgement [A, The manner, attitude, or words of the acupuncturist; B, The manner, attitude, or words of the assistant (outcome assessor); C, The sensation of the acupuncture stimulation; D, The results of the acupuncture treatment (e.g., changes in mood and sleep, etc.); E, The experience of the acupuncture procedure (e.g., what the acupuncturist did and how it felt)].

^*a*^Correct guess.

RA, real-acupuncture; SA, sham-acupuncture; CI, confidence interval.

The James' blinding index was 0.67, and the upper bound of the 95%CI was 0.75 (>0.5), suggesting there was insufficient evidence to show unblinding between the two groups. Furthermore, although two thirds of the participants (67%) in the RA group correctly guessed their group allocation, comparing to less than half (44%) in the SA group, the Bang's blinding index of the SA group was-−0.14 (−0.20 < Bang's blinding index cut-offs < 0.20), suggesting that participants in SA were also successfully blinded (Table 13). Taken together, we can assume that blinding was successful in our study and the efficacy-related outcomes thereby were credible.

## 4. Discussion

### 4.1. Summary of findings

To the best of our current knowledge, this is the first sham-controlled RCT to investigate acupuncture for comorbid depression and insomnia during perimenopause, and to assess the effect in the short-, medium-, and long- term. Blinding was successful and only minor AEs were reported. The RA group reported significantly reduced PSQI scores at post-treatment and 8-week follow-up, to a greater extent than the SA group, but did not differ from the SA group on either the reduction of HAM-D_17_ scores or changes in serum reproductive hormones. Overall, acupuncture is safe and contributes to clinically relevant improvements in insomnia among women with comorbid PMD and PMI, with satisfactory short- and medium- term effects.

### 4.2. Interpretation of findings and comparing with other studies

#### 4.2.1. Comparison with prior RCTs on PMD or PMI

Given no acupuncture trials examining the comorbid PMD and PMI, we compare our results with trials examining either PMD or PMI.

For PMD, two previous RCTs reported the benefits of electroacupuncture (2, 59). The absence of a placebo-control (placebo-/sham- acupuncture) group in both trials made it difficult to distinguish the real effect from that of placebo. Furthermore, those two RCTs did not clarify whether participants had comorbid insomnia or not, nor state if participants used hypnotics/sedatives at the entry or during the trial. For instance, Eszopiclone shows effects in improving PMD (60). They also did not report any sleep-related outcome measures (2, 59). As a consequence, the effectiveness of acupuncture in those trials might have been overestimated.

In agreement with our results, two previous RCTs confirmed the positive efficacy of acupuncture amongst patients with PMI (6, 36). One of them explicitly excluded patients with a diagnosis of depression, and only required participants aged 45–60 years old with amenorrhea for at least 6 months. Since a standard international guideline such as STRAW criteria (30) for the perimenopause diagnosis and patients screening was not used (36), some post-menopausal women may have been incorrectly included. Furthermore, Estazolam at variable dosages (1–2 mg) was allowed in that RCT, making it difficult to determine whether the sleep-promoting benefit was caused by acupuncture and/or the hypnotic (36). The other RCT employed manual acupuncture as we did, but only reported the short-term effect of acupuncture because it did not include any follow-up (6).

#### 4.2.2. Acupuncture has shown evidence for improving mood and sleep

The results of ITT and PP analyses in the current trial were consistent across most outcome measures, we therefore focus our discussion on ITT results.

From baseline to post-treatment, HAM-D_17_ scores decreased by 3.09 points in the RA group, and by 1.93 in the SA group. A 3-point difference on HAMD is viewed as the “minimal improvement” (61). Thus, RA-induced but not SA-induced amelioration on depressed mood is of clinical significance. Remission of depressive symptoms (reduction of HAM-D_17_ scores) in the SA group might be partially attributed to the basic perimenopausal mental health and sleep hygiene education delivered at the beginning of treatment and placebo effect. After all, a previous study has shown that Chinese perimenopausal women usually have a positive attitude toward acupuncture treatment and this high expectation is likely to over-optimize the responses (6). Using the data, we calculated the sample size needed to detect a difference of 1.16 on HAM-D_17_ was 116. Our sample size was too small. A reduction of at least three points in PSQI scores suggests a minimal clinically significant difference in sleep symptom (62). The PSQI scores was significantly reduced by 2.77 and 2.74 (approaching three points) in the RA group at post-treatment and 8-week follow-up, respectively. The PSQI reduction in the SA group was only 0.78 and 0.39, respectively. The changes in the RA group approached clinical significance whereas changes in SA are of no clinical relevance. The changes of Meno-D and ISI scores are broadly consistent with the changes of HAM-D_17_ and PSQI, respectively, supporting the aforementioned findings.

Despite satisfactory short- and medium- term efficacy, the long-term efficacy of acupuncture is not supported. In contrast, Li et al. reported that, for PMD, acupuncture had excellent longstanding anti-depressive efficacy, better than Escitalopram (2). This discrepancy between the two studies may be attributed to the different acupuncture stimulation as electroacupuncture was adopted in Li et al.'s trial, and manual acupuncture was used in the current trial.

Accumulated evidence demonstrated that stressful life events not only exacerbate somatic symptoms of menopause (63) but also contribute to both insomnia and mood disorders (63–66). We controlled this confounding factor by using SRRS and SCSQ to assess social adjustment and coping and found no difference between RA and SA groups.

The associations between depression and somatic symptoms, and sleep and anxiety are well-established, and PMD and PMI are susceptible to vasomotor symptoms, sexual dysfunctions, and/or muscles and joints pains (59, 67). The degree of the decrement in QoL is proportional to the severity of depressive symptoms (68). Similarly, compared with good sleepers, insomniacs report lower QoL, and report more somatic discomforts and emotional difficulties (69). In the current study, the changes in HAM-A scores indicated the advantage of RA outweigh SA on the remission of anxiety, but there is no difference between RA and SA on changes in menopausal symptoms and QoL as measured with KI and Men-QoL. This could be due to acupoints selection as our choice of acupoints focused on mental health and insomnia, but not on vasomotor symptoms, sexual drive or pain. However, an adequately powered RCT specifically examined acupuncture for moderate to severe menopausal hot flashes and did not show significant difference between RA and SA on either hot flushes or QoL (70).

#### 4.2.3. Insufficient evidence supports the effect underlying acupuncture being mediated through modulating hormone levels

Both PMD (71) and PMI (72) symptoms are closely associated with the fluctuations and changes in reproductive hormone levels. Oocyte depletion and ovarian aging result in the profound alterations at the biological levels, including fluctuating and in the end the decrements in E_2_ level and the increments in FSH and LH levels (73, 74). Ma et al. reported that acupuncture increased the serum E_2_ and decreased the LH and testosterone levels amongst women with menopause syndrome, accompanied with the improvements in a variety of perimenopausal symptoms (75). In consistence, three systematic reviews all suggested that acupuncture down-regulates serum FSH and LH levels and up-regulates serum E_2_ level in menopausal women, and was comparable to that of HRT (76–78). However, due to the low quality and limited number of original RCTs included, acupuncture's benefits on PMD/PMI links to the regulation of reproductive hormone remains undefined.

In the current trial, we found that acupuncture did not reverse the normal trend of hormonal changes over time. Our findings are consistent with those of two previous RCTs, reporting that electroacupuncture significantly ameliorated PMD but did not change hormone levels (2, 59). Several reasons might explain the conflicting results amongst the literature. First, the declines of ovarian follicular function and its induced changes in hormonal milieu and hormone levels during perimenopause is an irreversible physiological process (2). TCM theory also acknowledges that acupuncture is unlikely to stop or reverse this natural process (79). Instead, it helps women reach a new balanced state (23). Second, the pathogenesis of PMD/PMI is complex and associated with multiple causal factors, and is not entirely hormone based (80, 81). In our previous review, we proposed that the underlying mechanisms of acupuncture against PMD/PMI may also link to the modulation of neurotransmitters, oxidative stress, hypothalamic-pituitary-adrenal axis /hypothalamic-pituitary-ovary axis, various signaling pathways, and/or other cellular events (23). Third, Pimenta et al. linked the exacerbation of symptomatology in perimenopausal women to life conditions and events, rather than hormonal changes (82). Zhou et al. employed a structural equation model and reported that hormones might not be directly correlated with depression, while perimenopause-related symptoms and QoL can function as mediating variables for the path from hormones to depression (59). Finally, a survey targeting Chinese perimenopausal women confirmed that the effect of sleep quality on subjective wellbeing was partially mediated by their anxiety symptoms (83). In the current trial, the trend in anxiety was consistent with that in PMD/PMI, with a significant decline following RA treatment. The reduction in anxiety thereby can also partially explain the improvement in PMD and PMI.

In short, acupuncture-induced amelioration on PMD/PMI is likely to be achieved through multiple pathways, including normalizing the disordered physiological function, reducing anxiety, and improving QoL. Further research is warranted to elaborate the exact biological and/or psychosocial mechanisms underlying the therapeutic effects of acupuncture.

#### 4.2.4. Safety, tolerance, and satisfaction of acupuncture

*De-qi* sensation, an indicator that acupuncture achieves its ideal dosage, was reported by all participants in the RA group. All participants tolerated the treatment well and none withdrew from the trial due to needling sensation or bad experience of needling. In addition, the incidence and extent of AEs associated with acupuncture treatment were minimal and tolerable, and usually disappeared within a maximum of 72 h after treatment sessions. Those practical and safety data will be of interest to women as many of them are attracted to CAM including acupuncture for its safety profile (84).

We measured expectation (AES) at baseline and ninth treatment session, and satisfaction (MS-TSQ) at end of ninth and seventeenth sessions. It is interesting to note a decline in expectation of acupuncture treatment from baseline to ninth session in both RA and SA groups; yet the satisfaction level increased in the RA group but decreased in the SA group by end of seventeenth session. The between-group difference in satisfaction is consistent with that observed in PSQI, reflecting the effect of RA continues to increase after tenth treatment session, and patients should be encouraged to complete the full course of acupuncture treatment.

### 4.3. Strengths and limitations

There are a few strengths of the current trial. First, to ensure we recruited women with similar presentation, we not only selected perimenopausal women with comorbid PMD and PMI, but also limited recruitment to one specific type of TCM syndrome diagnosis “Depression of *Liver* and Deficiency of *Kidney*,” which is the most common pattern of presentation confirmed by bibliometric studies (23). This design mirrors the clinical scenario, in which treatment regimen should be tailored to individual syndrome pattern or syndrome cluster (70). By limiting to people with one specific syndrome pattern we were able to semi-standardize our trial treatment and to enhance internal validity. Second, we used a SA procedure that is invasive with minimal needle manipulation to acupoints that have no specific effect on either insomnia or depression (35) to enhance the blinding. This SA design has been used in an acupuncture trial for insomnia with successful blinding of participants (85), and it was verified with both James' and Bang's blinding indices in our trial. This choice of SA is due to many Chinese women would have some knowledge about acupuncture. Non-invasive needling to non-acupoints would compromise the blinding. Third, it was thought that female estrogen might explain the acupuncture effect on PMI (6, 36). Our study was the first to examine this hypothesis and found reproductive hormone changes do not explain the improvements. Finally, we applied ITT and PP analyses and found the results were consistent, suggesting the robustness of the findings.

Due to its preliminary nature, some limitations of this trial should be acknowledged. First, larger than anticipated cases lost to follow-up forced us to use multiple imputation approach to replace the missing data. Started in March 2022 and lasted for more than 3 months, Shanghai witnessed the China's worst outbreak of SARS-CoV-2 virus since early 2020 (86). Seven participants declined further follow-ups due to this sudden outbreak, and reported that the pandemic exacerbated their mood and sleep disturbances. It is undeniable that there might be subtle differences between these software-generated data and the real experience of the participants. However, such difference does not appear to significantly impact on our findings, given the results of ITT and PP were consistent in all the key outcome measures. Second, the long-term efficacy of acupuncture might have been somewhat underestimated because five participants took part in the 16-week follow-ups during this strong wave of pandemic. It is likely they too experienced worsened sleep or mood during this time as those who dropped out during this period. Third, whilst self-reported sleep has improved following RA intervention, if and how acupuncture might change sleep process and architecture is unknown as we did not include PSG in the trial. We made this decision due to previous experience at the study center. After the initial Wuhan outbreak of COVID-19, almost all participants who had been interested in sleep clinical trials conducted at our center declined in-hospital PSG sleep monitoring. Considering the participants' willingness and the trial's feasibility, we did not include PSG in the current RCT. Fourth, without PSG, the screening of other sleep disorder, such as sleep apnea or restless leg syndrome, was based on trial doctor's thorough inquiry, diagnostic criteria for sleep disorder (i.e., DSM-V and ICSD-3), and participants' medical history; it is therefore possible some participants had more than PMI. This is, however, not likely to affect our current conclusion regarding the application of acupuncture to women with simple PMI. Comorbid insomnia and other sleep disorders can only be more complex and difficult to treat than simple insomnia. Even if the included participants incorrectly included those with comorbid PMI and other sleep disorders, we have affirmed the sleep-promoting effect of acupuncture in them. Fifth, participants with severe depressive episode/insomnia or other serious physical/mental illness were excluded at screening stage. Our findings thereby cannot be generalized to sicker patients, or perimenopausal women accompanied with other complex/severe comorbidities.

### 4.4. Implications for research and clinical practice

The current trial has provided essential data on safety, acceptance and effectiveness of acupuncture for comorbid PMI and PMD. Future trials may consider a wait-list control, expanding the sample sizes, and extending the follow-up period, to confirm the current findings and clarify whether the remission in depression is a specific effect of acupuncture or only a mega-placebo or natural regression. It is also necessary to examine when acupuncture is combined with sedating-antidepressant agent if it can further augment anti-depressive/sleep-promoting effects or further reduce medication-induced AEs. Collecting the information regarding participants' previous acupuncture experience is also encouraged in future study since this experience may affect their acceptability and compliance.

Clinically, comorbid PMI and PMD may require multidisciplinary approaches (8, 9, 23), and acupuncture could be an ideal adjunct therapy for insomnia and depression. A sufficient number of acupuncture treatment sessions is required for the optimal effect. Given we were interested in assessing the short-term, medium term and long-term effects of acupuncture and did not want to burden participants with data collection at multiple points, we did not track participants' weekly or monthly changes in PMI and PMD during the 8-week treatment. We therefore cannot comment on the progression of participants or the accumulative effect of acupuncture. Future studies may look into the weekly changes immediately after acupuncture treatment to gain a better understanding on this aspect.

## 5. Conclusions

Seventeen sessions of acupuncture treatment provided amelioration of both PMD and PMI symptoms for eight-weeks after termination of treatment. The specific anti-depressive effect of acupuncture is yet to be determined. The longstanding effect of acupuncture is suboptimal. Given serum hormone levels were not altered by acupuncture, the mechanism of action is likely not to be due to changes in reproductive hormones. Acupuncture represents a safe and useful non-pharmacologic intervention option for women with mild to moderate comorbid PMD and PMI, particularly those who are intolerant to or reluctant to receive HRT.

## Data availability statement

The original contributions presented in the study are included in the article/Supplementary material, further inquiries can be directed to the corresponding authors.

## Ethics statement

This study involving human participants were reviewed and approved by Human Research Ethics Committee (HREC) of the Shanghai Municipal Hospital of Traditional Chinese Medicine, Shanghai University of Traditional Chinese Medicine, Shanghai, China; and endorsed by the HREC of RMIT University, Melbourne, Australia. The patients/participants provided their written informed consent to participate in this study.

## Author contributions

ZZ, W-JZ, and F-YZ conceived and designed this trial. RC and GK contributed to the trial design and guidance in analysis methods. W-JZ and H-RW were responsible for participant recruitment. HX was in charge of delivering treatment. F-YZ and Y-LH were responsible for outcome assessments and data collection. TJ conducted randomization and allocation concealment. Data analysis was performed by Q-QF and F-YZ. The manuscript was drafted by F-YZ and was carefully revised and edited by ZZ, RC, GK, and W-JZ. All authors have read this manuscript and approved the submitted version.
