# Supplementary material for: Acupuncture for comorbid depression and insomnia in perimenopause: A feasibility patient-assessor-blinded, randomized, and sham-controlled clinical trial
Source: Front Public Health. 2023 Feb 6;11:1120567. doi: 10.3389/fpubh.2023.1120567 (PMC9939459; doi:10.3389/fpubh.2023.1120567)
Supplement: Supplementary file 1 [file Data_Sheet_1.pdf]

Supplementary materials

Appendix 1 Diagnostic criteria for "Depression of *Liver* and Deficiency of *Kidney*" pattern

**Symptoms**	**Descriptions**
Primary symptoms	depressed mood, fatigue, chest distress and preference for sighing, insomnia or dreamful sleep, hot flashes and sweating, women with menstrual disorders, low menstrual flow or menopause, red tongue with little coating, string and thready pulse or string and rapid pulse
Secondary symptoms	frequent urination; dribbling urination
	distending pain of breasts
	headache; fullness in head
	hypochondrium distress and/or pain
	soreness and weakness of waist and knees or heel pain
	dysphoria
	feverish feeling in palms and/or soles
	blurred vision
	dizziness and tinnitus
	spontaneous sweat
	memory loss
	hyposexuality
	thirsty
Diagnosis criteria: meet all the primary symptoms with or without secondary symptoms can be diagnosed as insomnia with TCM pattern of “Depression of *Liver* and Deficiency of *Kidney*”	

Appendix 2 Suggestion list for mental health and sleep hygiene in perimenopause

1. Correctly understand that perimenopause is a normal physiological process, and some of the symptoms that you experience are caused by sex hormone fluctuations. After a period of time, you will gradually adapt perimenopause
2. Have regular life and rest, and not to stay up late
3. Have a balanced diet; do not eat food that is too spicy, too cold, greasy, salty or too sweet
4. Try to maintain a positive mood
5. Actively participate in entertainment, exercise and social activities
6. Maintain a moderate sex life
7. Keep a healthy sleep habit (e.g., don't use your electronic products while lying in bed, keep a calm mood half an hour before going to bed, including not watching exciting movies or TV shows, or chatting excitedly with family or friends, don't eat anything or do vigorous physical activity within 2 hours before going to bed, etc.)
8. Harmonize with the relationship between yourself and family, colleagues, and friends
9. Regular physical examinations, especially the examinations of gynecological, breast and endocrine
10. Do not bring the problem to bed, leave the problem for tomorrow (do not think about the problem or make plans while lying in bed)

Appendix 3 Main indications of each selected acupoint in the RA/SA group

Groups	Acupoints	Main indications according to "*Indications of acupoints (National Standard of People's Republic of China, GB/T 30233-2013)*"
RA group	CV4	Hernia; abdominal pain; urinary retention; spermatorrhea; impotence; irregular menstruation; dysmenorrhea; lochiorrhea; uterine prolapse; infertility; diarrhea; fatigue
	CV7	Hernia; abdominal pain; irregular menstruation; infertility; postpartum disorder; urinary retention; edema
	EX-CA1	irregular menstruation; dysmenorrhea; infertility; uterine prolapse
	EX-HN3	mental and psychological disorders
	GV20	mental and psychological disorders; head/face diseases; anal prolapse and hysteroptosia
	PC6	mental and psychological disorders; cardiac diseases; palpitations; chest tightness; stomachache; vomiting; hiccups; upper extremity pain
	KI3	gynecological disorders; genitourinary system disorders; lower extremity paralysis; respiratory system disorders
	LR3	gynecological disorders; genitourinary system disorders; lower extremity paralysis; sore throat
	SP6	gynecology disorders; genitourinary system disorders; digestive system disorders
SA group	GB38	waist and lower extremities pain/limited mobility; chest pain; sore throat
	GB39	waist and lower extremities pain/limited mobility; inappetence and distention
	LI12	elbow and arm pain, numbness or paralysis
	LI13	elbow and arm pain; tuberculosis of lymph nodes
	ST38	lower extremity paralysis
	TE8	deafness; hoarseness; toothache; upper extremity pain
	TE9	deafness; toothache; upper extremity pain

Abbreviations: RA, real-acupuncture; SA, sham-acupuncture; CV4, Guanyuan; CV7, Yinjiao; EX-CA1, Zigong; EX-HN3, Yintang; GB38, Yangfu; GB39, Xuanzhong; GV20, Baihui; KI3, Taixi; LI12, Zhouliao; LI13, Shouwuli; LR3, Taichong; PC6, Neiguan; SP6, Sanyinjiao; ST38, Tiaokou; TE8, Sanyangluo; TE9, Sidu

Appendix 4 Sleep hygiene behavior/habit checklist (SHBC)

Items	Yes (1 point)	No (0 point)
1. Stay up late; or Do not have regular sleep routine		
2. Do not have a good sleep environment (e.g., you will be disturbed by light and/or sound, or cannot avoid extreme temperature and/or humidity while sleeping, etc.)		
3. Do not have a balanced diet; or Eat food that is too spicy, too cold, greasy, salty or too sweet		
4. Drink strong tea or caffeinated or alcoholic beverages after 12 P.M.		
5. Take naps at daytime except at lunch break; or The nap time at lunch break more than 30 minutes		
6. Cannot maintain a positive mood		
7. Watching exciting movies or TV shows, or chatting excitedly with family or friends half an hour before going to bed; or Eat food or do vigorous physical activity within 2 hours before going to bed		
8. Use your electronic products (e.g., phone, pad, etc.) while lying in bed		
9. If you wake up or go to the toilet at midnight, you often check the time before lie down and continue to sleep		
10. Often bring the daily problems to bed; or Often make plans while lying in bed		
Total scores		

Appendix 5 Schedule of enrolment, interventions, and assessments

Timepoints	Enrolment -1 week	Baseline assessment	Intervention phase		Post-treatment assessment	Follow-up assessment	
			0 day	8 week		8th week	16th week
Enrolment							
Eligibility screen	X						
Informed consent	X						
Medical history	X						
Physical examination	X				X		X
Allocation	X						
Interventions							
RA			17 sessions of RA treatment on relevant acupoints to PMD/PMI				
SA			17 sessions of SA treatment on irrelevant acupoints to PMD/PMI				
Outcome measures							
Primary outcome							
$HAM\text{-}D_{17}$		X			X	X	X
PSQI		X			X	X	X
Secondary outcomes							
Meno-D		X			X	X	X
ISI		X			X	X	X
KI		X			X	X	X
MenQoL		X			X	X	X
HAM-A		X			X	X	X
Sex hormone level test (FSH, E_2, LH)		X			X		
SHBC		X			X		X
Impact of and responses to stressful life events							
SRRS		X					X
SCSQ		X			X		X
Expectation, sensation, perception and satisfaction of acupuncture							
AES		X	end of ninth treatment session				
MASS			end of ninth treatment session				
APS					X		
MS-TSQ			end of ninth treatment session		X		
Adverse events		X	X	X	X	X	X
Success of blinding							X

Abbreviations RA, real-acupuncture; SA, sham-acupuncture; $HAM\text{-}D_{17}$, 17 items-Hamilton Depression Rating Scale; PSQI, Pittsburgh Sleep Quality Index; ISI, Insomnia Severity Index; KI, Kupperman Index; MenQoL, Menopause-specific Quality of Life; HAM-A, Hamilton Anxiety Scale; SHBC, Sleep Hygiene Behavior Checklist;

SRRS, Social Readjustment Rating Scale; SCSQ, Simplified Coping Style Questionnaire; AES, Acupuncture Expectancy Scale; MASS, Massachusetts General Hospital Acupuncture Sensation Scale; APS, Acupuncture Perception Scale; MS-TSQ, Menopause Symptoms Treatment Satisfaction Questionnaire; FSH, follicle-stimulating hormone; E_2, estradiol; LH, luteinizing hormone.

Appendix 6 Main effect, time effect, and interaction effect analysed by linear mixed-effect models for HAM-D_{17}, PSQI, Meno-D, ISI, KI, MenQoL, HAM-A, SHBC and SCSQ outcomes with ITT/PP analysis (ITT dataset, participants = 70; PP dataset, participants = 41)

Outcome	Analysis method	N	Main effect		Time effect		Interaction (time*group) effect	
			F	*p value*	*F*	*p value*	*F*	*p value*
HAM-D_{17}	ITT	70	0.56	0.46	16.15	< 0.01	0.81	0.37
	PP	41	0.09	0.77	10.93	< 0.01	0.67	0.57
PSQI	ITT	70	2.00	0.16	12.98	< 0.01	4.56	0.33
	PP	41	5.03	0.03	11.94	< 0.01	5.91	< 0.01
Meno-D	ITT	70	0.46	0.50	23.58	< 0.01	0.37	0.54
	PP	41	0.20	0.66	14.58	< 0.01	0.12	0.95
ISI	ITT	70	2.72	0.10	12.03	< 0.01	4.55	0.03
	PP	41	5.22	0.03	12.16	< 0.01	6.30	< 0.01
KI	ITT	70	0.33	0.57	12.37	< 0.01	0.04	0.84
	PP	41	0.12	0.73	8.12	< 0.01	0.45	0.72
MenQoL								
Vasomotor	ITT	70	0.01	0.94	0.26	0.85	0.26	0.61
	PP	41	0.78	0.38	0.27	0.85	0.93	0.43
Psychosocial	ITT	70	0.10	0.76	18.51	< 0.01	0.80	0.37
	PP	41	0.07	0.80	17.49	< 0.01	0.85	0.47
Physical	ITT	70	0.50	0.48	4.66	< 0.01	0.33	0.57
	PP	41	0.05	0.83	4.73	< 0.01	0.31	0.82
Sexual	ITT	70	0.05	0.82	0.04	0.96	0.31	0.58
	PP	41	0.49	0.49	1.30	0.28	5.05	< 0.01
HAM-A	ITT	70	1.68	0.20	5.47	< 0.01	2.64	0.10
	PP	41	3.73	0.06	4.44	< 0.01	2.96	0.04
SHBC	ITT	70	0.02	0.90	38.73	< 0.01	0.08	0.78
	PP	41	0.47	0.50	28.85	< 0.01	0.88	0.42
SCSQ								
positive coping	ITT	70	0.52	0.47	0.38	0.69	0.37	0.55
	PP	41	0.01	0.92	1.61	0.21	0.16	0.85
negative coping	ITT	70	3.39	0.07	1.10	0.36	0.28	0.59
	PP	41	1.91	0.18	2.30	0.11	0.31	0.74

Abbreviations ITT, Intention-To-Treat analysis; PP, Per-Protocol analysis; HAM-D_{17}, 17 items-Hamilton Depression Rating Scale; PSQI, Pittsburgh Sleep Quality Index; ISI, Insomnia Severity Index; KI, Kupperman Index; MenQoL, Menopause-specific Quality of Life; HAM-A, Hamilton Anxiety Scale; SHBC, Sleep Hygiene Behavior Checklist; SCSQ, Simplified Coping Style Questionnaire.
